# Edoxaban: front-line treatment for brachiocephalic vein thrombosis in primitive mediastinal seminoma: A case report and literature review

**DOI:** 10.1097/MD.0000000000029429

**Published:** 2022-08-26

**Authors:** Agnese Maria Fioretti, Tiziana Leopizzi, Agata Puzzovivo, Francesco Giotta, Vito Lorusso, Giovanni Luzzi, Stefano Oliva

**Affiliations:** a Cardio-Oncology Unit, IRCCS Istituto Tumori Giovanni Paolo II, Viale Orazio Flacco 65, 70124, Bari, Italy; b Cardiology-Intensive Care Unit, Ospedale SS. Annunziata, Via Francesco Bruno 1, 74121, Taranto, Italy; c Medical Oncology Unit, IRCCS Istituto Tumori Giovanni Paolo II, Viale Orazio Flacco 65, 70124, Bari, Italy.

**Keywords:** cancer-associated thrombosis, chemotherapy, direct oral anticoagulant, edoxaban, upper limb deep vein thrombosis, low molecular weight heparin, seminoma

## Abstract

Venous thromboembolism is a feared frequent complication of cancer with a 2-way relationship. Low molecular weight heparin is the mainstay of treatment. The use of direct oral anticoagulants is supported by established evidence for the treatment of deep vein thrombosis also in active cancer and they are prioritized over low molecular weight heparin for cancer-associated thrombosis according to current guidelines. However, upper limb deep vein thrombosis is poorly studied with scant data on the use of direct oral anticoagulants in noncatheter-related deep vein thrombosis. We report the case of a patient with noncatheter-related deep vein thrombosis and a rare tumor site effectively and safely treated with a direct oral anticoagulant, edoxaban, after lack of efficacy with low molecular weight heparin.

**Patient concerns:**

A 35-year-old man with primitive mediastinal seminoma presented at our Cardio-Oncology Unit for prechemotherapy assessment.

**Diagnosis:**

Persistent brachiocephalic deep vein thrombosis, despite full-dose enoxaparin, was detected at ultrasonography.

**Intervention:**

We decided to switch the anticoagulant treatment from enoxaparin to edoxaban.

**Outcome:**

The 3-month ultrasonography showed almost total regression of the deep vein thrombosis without any adverse effects and a good patient compliance.

**Lessons:**

We conducted a literature review on upper limb deep vein thrombosis, since its management is challenging due to inconsistency of evidence. This report highlights the benefits of direct oral anticoagulants compared to low molecular weight heparins in cancer-associated thrombosis therapy in terms of efficacy, safety and ease of use.

## 1. Introduction

Thromboembolism is the second leading cause of death among cancer patients after progression of the disease itself,^[1–5]^ contributing to increased morbidity and mortality.^[6]^ Furthermore, venous thromboembolism (VTE) is the first manifestation of a hidden cancer.^[7]^ Although published guidelines have provided indications for the management of cancer-associated thrombosis (CAT), limited data still exist on special populations such as patients with noncatheter-related upper limb deep vein thrombosis (nonCA-ULDVT),^[8]^ thus making its management particularly challenging. Consequently, we report a clinical case of a young male patient with primitive mediastinal seminoma undergoing treatment with bleomycin, etoposide and cisplatin (BEP Protocol) who presented with marked upper limb deep vein thrombosis (ULDVT) unresponsive to low molecular weight heparin (LMWH). Anticoagulant therapy was switched to edoxaban, a direct oral anticoagulant (DOAC), which was able to dissolve the extended deep vein thrombosis (DVT), suggesting that DOACs may be a reliable option especially in long-term anticoagulant treatment and also in patients with nonCA-ULDVT as in this case. A literature review on CAT and the use of DOACs in ULDVT was added. Pubmed, Embase and Cochrane were searched up to December 2021 with the following key words: “upper limb vein thrombosis,” “upper extremity vein thrombosis,” “axillary vein thrombosis,” “brachial vein thrombosis,” “internal jugular vein thrombosis,” “subclavian vein thrombosis,” “cancer,” “edoxaban,” “direct oral anticoagulant,” and “low molecular weight heparin.”

## 2. Case presentation

**Figure 1.**
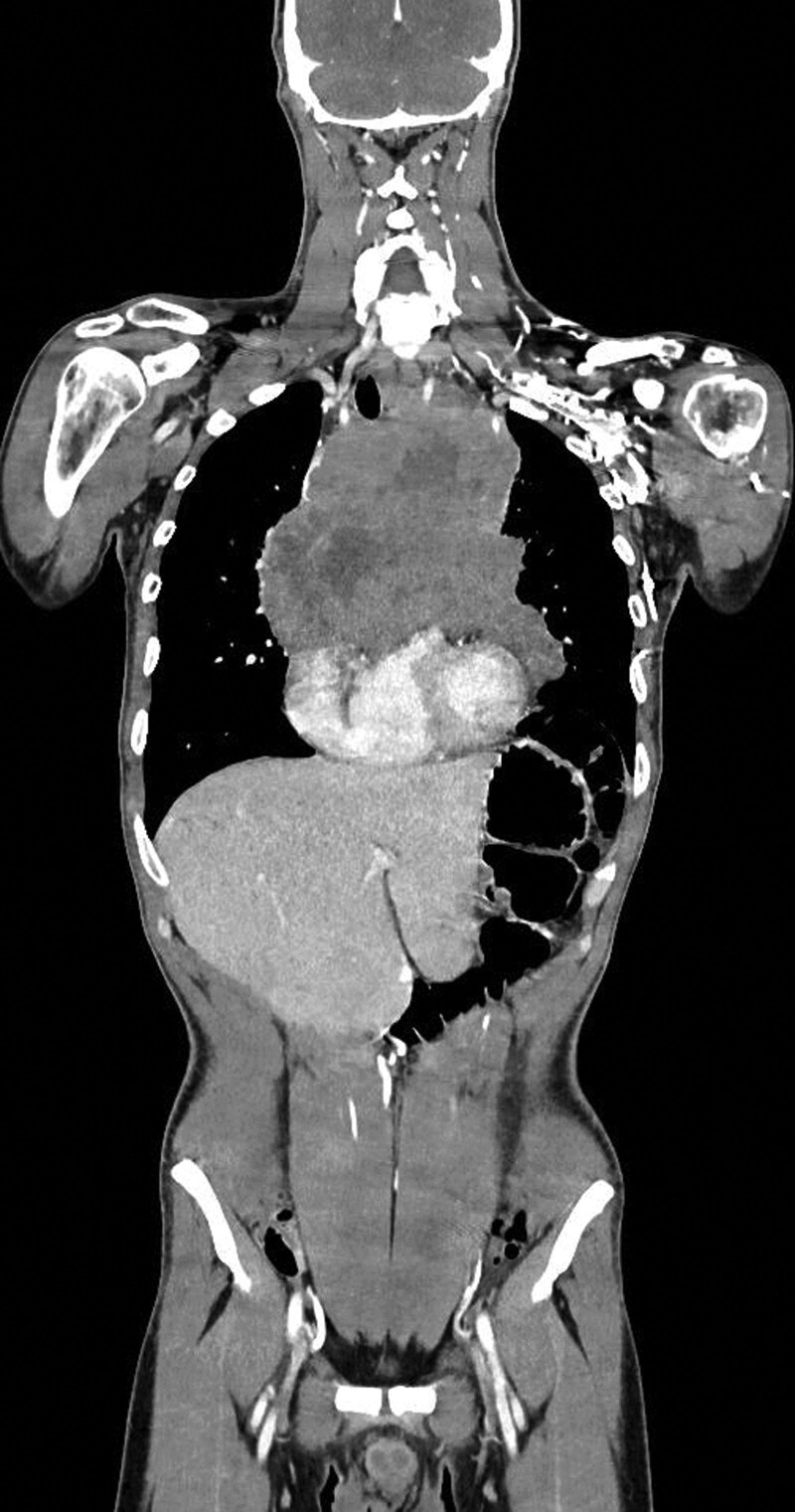
CT scan (coronal and axial axis) showing a solid antero-superior mediastinal vascularized mass (16 × 13 cm) encasing the great thoracic vessels.

**Figure 2.**
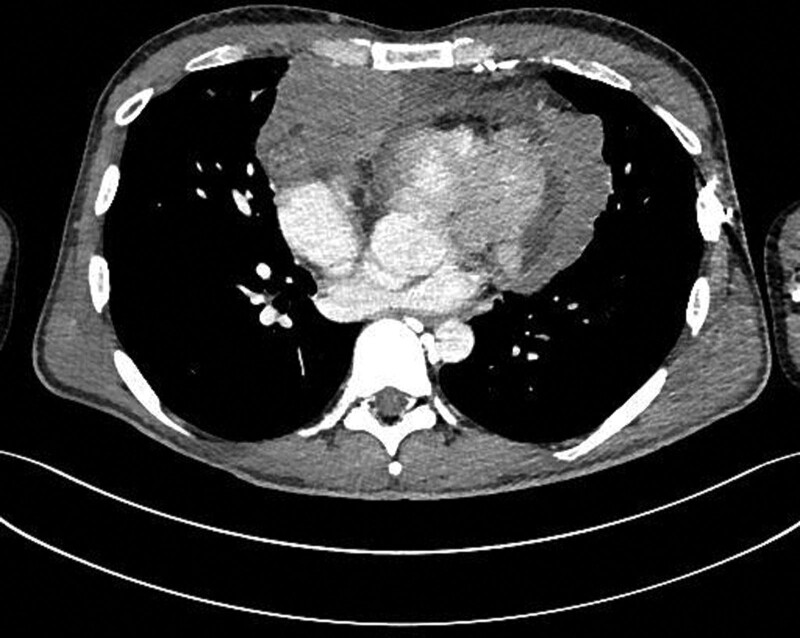


**Figure 3.**
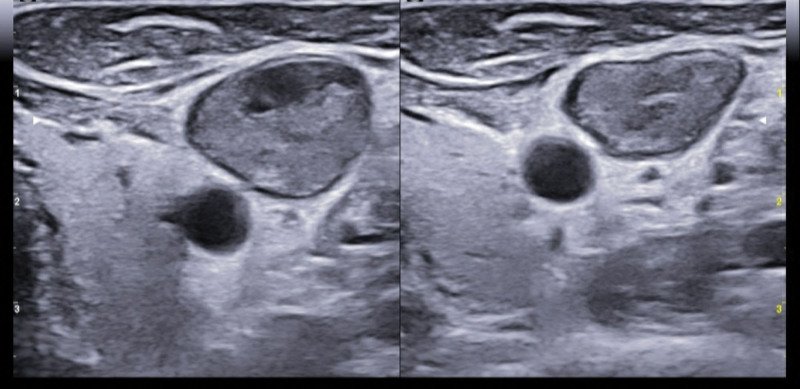
Ultrasonography baseline-images: short axis view of the left jugular vein (compression ultrasound CUS maneuver) and long axis view of the left jugular vein and subclavian vein, overall showing deep vein thrombosis.

**Figure 4.**
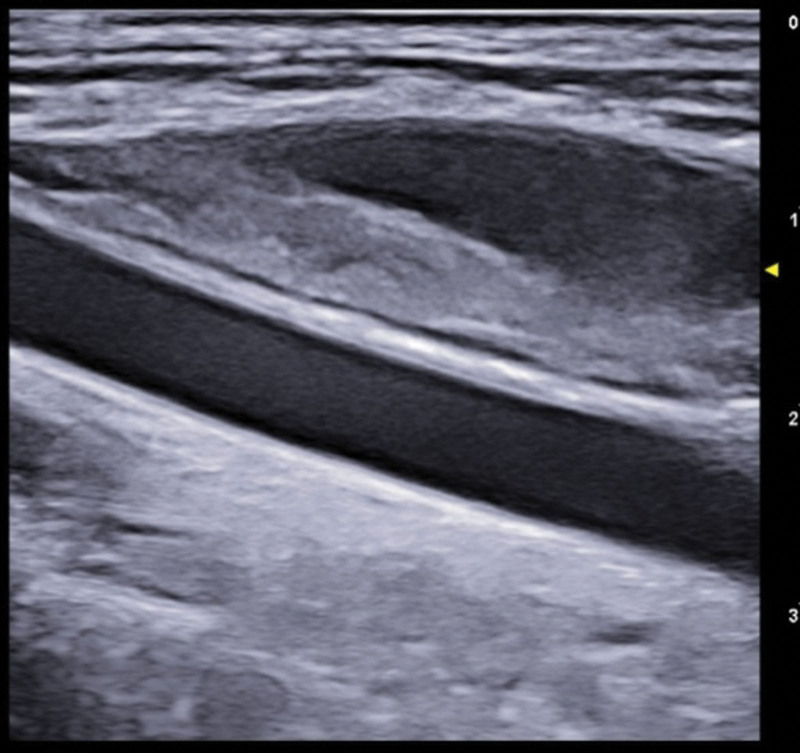


**Figure 5.**
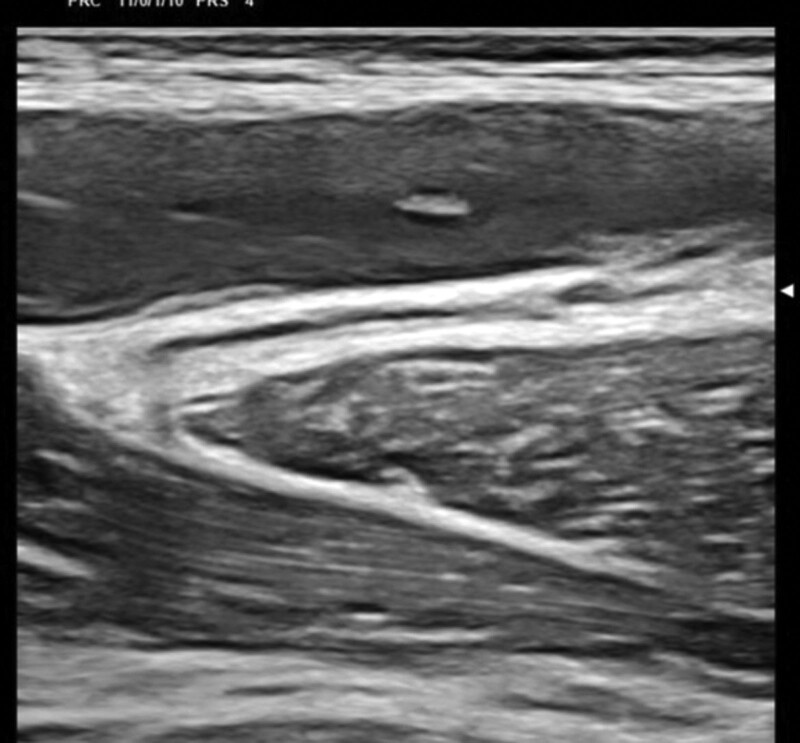


A 35-year-old Caucasian male, with an uneventful clinical history, presented with dysphagia and odynophagia associated with weight loss of about 15 kg in the previous 2–3 months. Esophagogastroduodenoscopy showed multiple ulcers in the middle third of the esophagus, esophagitis, cardias incontinence and chronic superficial gastritis. The esophageal biopsy and immunology panel were negative. Therapy with esomeprazole 40 mg/die was initiated. A thyroid ultrasonography (US) was also performed and, although negative, revealed an incidental thoracic mass compressing the proximal borders of the gland. Epiaortic US detected left DVT of the internal jugular (IJ), subclavian, axillary and brachial veins. Accordingly, LMWH therapy with enoxaparin 100 IU/kg twice-daily was administrated. A PET-CT scan showed intense pathological ^18^F-FDG uptake in the antero-superior mediastinum, suggestive of malignancy, notably in the left hemithorax. Further CT-scans found a huge solid antero-superior mediastinal vascularized mass (16 × 13 cm) encasing the thoracic great vessels with 20 cm longitudinal cranio-caudal extension and tracheal dislocation (Figs. 1–2). A lung perfusion-scan showed the total. Add absence of perfusion in the left lung. Arterial blood gas analysis on room air was performed (ph: 7.51, pCO_2_: 33 mm Hg, pO_2_: 61 mm Hg, HCO_3_: 26.3 mmol/L). CT-angiography showed critical compression of the trunk and both branches (especially the left one) of the pulmonary artery. The patient reported marked asthenia and sweating followed by presyncope. D-dimer: 6026 µg/L, NT-proBNP: 1417 pg/mL, Beta-hCG: 33.7 mlU/ml, Alpha-fetoprotein: 2.4 ng/ml, LDH: 1882 U/L, NSE: 85, CEA negative, β2microglobulinemia: 2.26. Testicular US was normal. The patient was thus referred to our Cardio-Oncology Unit. Physical examination was normal. Electrocardiogram revealed sinus tachycardia at 114 beats/min. Blood pressure was 120/80 mm Hg. Transthoracic echocardiography (TTE) revealed a periaortic cuff coming from the mediastinal mass, causing a compression from the outside of the trunk and both branches of the pulmonary artery with an almost total occlusion of the left branch. TTE also detected dilatation of the right heart chambers, ectasia of the supra-hepatic veins and the inferior vena cava (diameter: 21 mm) with decreased inspiratory collapse (< 50%) and pulmonary hypertension (systolic arterial pulmonary pressure: 52 mm Hg). Left ventricular function was preserved (ejection fraction: 55%). A mediastinal biopsy demonstrated seminoma (ki67+: 65%) positive for PLAP, CD117, CKAE1/AE3 (dot-like) and CK8-18 (dot-like), positive (aberrant) for CD10 and TdT, negative for IRF-4, PAX5, ALK, synaptophysine, TTF1, CD20,S100, SOX10, NUT1, CD34, CD45LC, MPO, p63, CD3, CDS, Cyclin D1 and CD30. After placement of a right femoral vein indwelling catheter, chemotherapy with the BEP Protocol (etoposide 100 mg/m^2^/day D 1-5, cisplatin 20 mg/m^2^/day D 1-5, bleomycin 30 units/day, day 2, 9, 16, every 3 weeks) was started. US confirmed DVT of the left brachiocephalic vein with “slow venous flow” and noncompressibility at the compression ultrasound (CUS) maneuver (Figs. 3–5). After 4 months of therapy with full dose enoxaparin, due to the persistence of extended DVT, it was replaced with a full dose DOAC, edoxaban 60 mg/die.

The 2-month follow-up US showed complete resolution of the brachiocephalic DVT with minimal residual left internal jugular deep vein thrombosis (IJDVT). D-dimer: 1554 µg/L, Beta-hCG < 0.1 mlU/ml.

**Figure 6.**
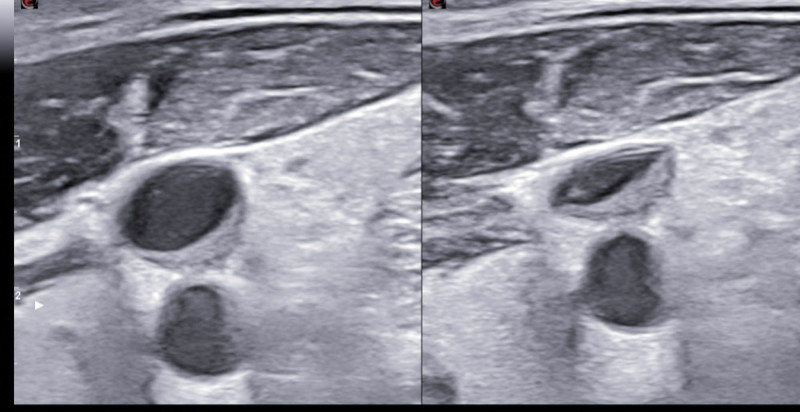
Ultrasonography 1-year follow-up images: short axis view of the left jugular vein (compression ultrasound CUS maneuver) and long axis view of the left jugular vein and subclavian vein, showing residual slight thrombosis only of the left jugular vein.

**Figure 7.**
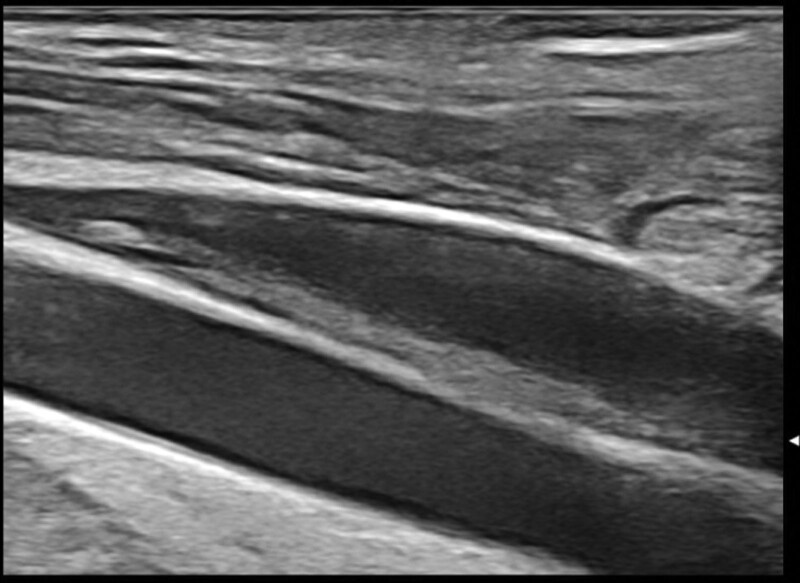


**Figure 8.**
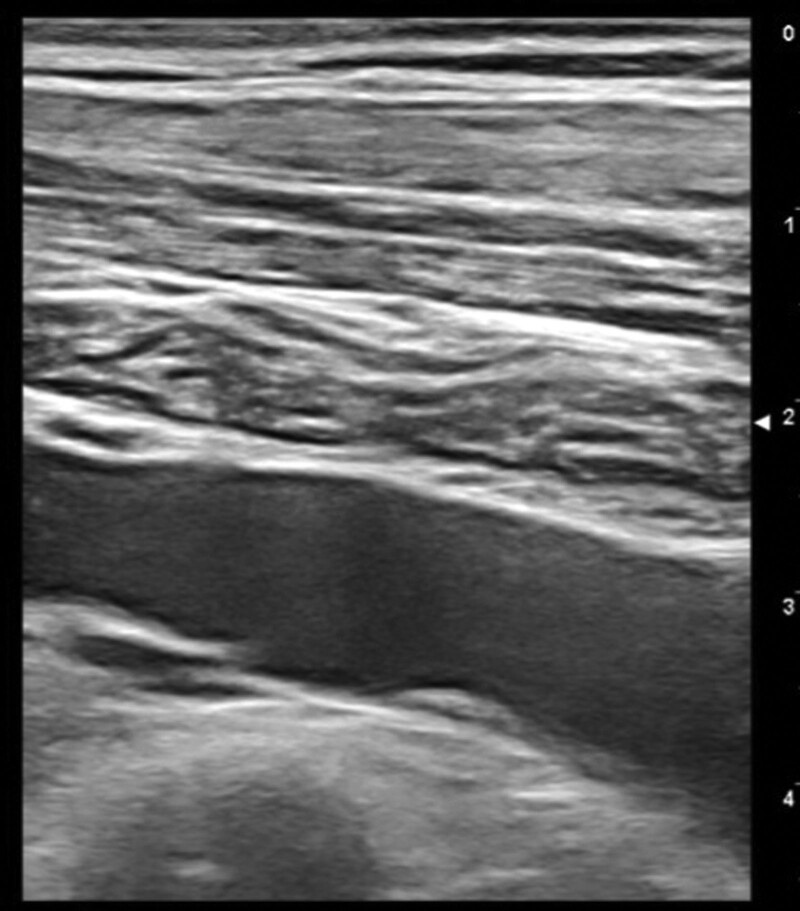


At completion of the BEP chemotherapy, lasting 4.5 months, a follow-up CT-scan showed that the mass had dramatically reduced (6 × 12 cm) and the normal anatomy of the thoracic great vessels and the trachea had been restored. The follow-up PET-CT scan detected a marked drop in mediastinal ^18^F-FDG uptake, indicating the efficacy of the completed anticancer regimen. 3 months after terminating the BEP Protocol, the patient started Proton therapy that lasted 1 month. At 12-month follow-up, US documented persistent slight left IJDVT without any adverse effects in therapy with edoxaban 60 mg 1 cp/die (Figs. 6–8). The patient was fully compliant and willingly accepted the extended treatment with edoxaban, due to the absence of adverse effects and the ease of administration (oral route rather than the previous daily self-injections of enoxaparin and the once-daily dose of the edoxaban pill).

## 3. Discussion

CAT is a common cause of morbidity and mortality in active cancer.^[9–11]^ Cancer patients are more likely to develop VTE than people without cancer, including DVT and pulmonary embolism (PE).^[12]^ The components of Virchow Triad (stasis, endothelial injury, and hypercoagulability) give rise to a greater risk of thrombosis in cancer patients.^[13]^ Overall, the primary site of cancer, the presence of metastatic disease, anticancer drugs, hormonal therapy, surgery, erythropoiesis-stimulating agents, and indwelling catheters contribute to VTE occurrence.^[14]^

The most endorsed and validated predictive model for CAT is the Khorana Risk Score (KRS). It encompasses clinical and laboratory variables (site of cancer, prechemotherapy platelet count, hemoglobin level, prechemotherapy leukocyte count and body mass index BMI).^[15]^ To improve the discriminatory performance of the Khorana Risk Score, other authors have proposed modifications by adding biomarker measurements (Vienna CATS) or type of chemotherapy (Protecht Risk Score) or by replacing BMI with performance status (CONKO Score).^[16]^ In particular, the Protecht Risk Score indicates that anticancer drugs contribute to the risk of VTE. Indeed, in calculating the VTE risk score, it assigns 1 extra point to patients receiving gemcitabine and/or platinum-based therapy.^[17]^

Currently, LMWH is the standard of treatment for CAT according to 2 large randomized clinical trials (RCTs) which compared warfarin with LMWH administration for 6 months in active cancer. Dalteparin significantly reduced VTE recurrence by 52% in 672 patients without influencing the rates of major bleeding and mortality compared to warfarin.^[18]^ In 900 patients, tinzaparin did not significantly reduce the risk of VTE recurrence, did not affect major bleeding or mortality, but significantly reduced nonmajor bleeding compared to warfarin.^[19]^ Recently, data from 4 head-to-head RCTs comparing DOACs to a LMWH, dalteparin, were published supporting the use of DOACs in CAT. The HOKUSAI-VTE CANCER trial was an open-label RCT that compared 6 to 12 months of the once-daily oral factor Xa inhibitor, edoxaban, versus dalteparin in symptomatic or incidental VTE in 1050 patients with cancer under active treatment. Edoxaban was not inferior to dalteparin as regards composite recurrent VTE or major bleeding (12.8% vs 13.5%). Recurrent VTE was reduced by edoxaban compared with dalteparin (7.9% vs 11.3%), but major bleeding increased (6.9% vs 4%), mostly due to higher bleeding rates in patients with gastrointestinal cancers (13.2% vs 2.4%).^[20]^ The SELECT-D pilot trial was an open-label RCT of 406 patients with active cancer and VTE treated for 6 months. Rivaroxaban reduced the risk of recurrent VTE compared with dalteparin (4% vs 11%) but increased the risk of clinically-relevant nonmajor bleeding (13% vs 2%).^[21]^ Further insights into DOACS safety in CAT treatment came from 2 RCTs comparing apixaban with dalteparin in active cancer patients. The ADAM-VTE trial studied 300 patients for 4 months. The primary outcome was major bleeding. Apixaban reduced recurrent VTE rates (0.7% vs 6.3%), major bleeding and clinically relevant nonmajor bleeding rates (6% for both groups). Major bleeding occurred in 0% of 145 patients receiving apixaban compared with 1.4% of 142 patients receiving dalteparin.^[22]^ The CARAVAGGIO trial is the largest (N: 1168) open-label, multicenter, non inferiority 6-month study comparing apixaban with dalteparin for CAT treatment. Apart from the efficacy and safety profile of DOACs in this clinical setting, assessment included patient-reported outcomes such as treatment satisfaction and pain. Patients treated with apixaban resulted 3-fold more compliant compared to patients receiving dalteparin. Apixaban reduced VTE recurrence compared with dalteparin (5.6% vs 7.9%) and, notably, with no increase in the rates of major bleeding (3.8% vs 4%).^[23]^ The updated meta-analysis of RCTs assessing the efficacy and safety of DOACs versus LMWH (dalteparin) in CAT showed that VTE recurrences were significantly reduced and rates of major bleeding were not significantly different between patients receiving DOACs and those treated with dalteparin.^[24]^ The study population comprised 2894 patients with cancer, the majority of whom received cancer agents and had metastatic disease, making the study groups representative of clinical practice. Most major bleeding events occurred in the gastrointestinal tract (58%). An increased risk of major bleeding in patients with gastrointestinal cancer was observed in the HOKUSAI-VTE CANCER and in the SELECT-D studies, but not in the ADAM-VTE and in the CARAVAGGIO trials. Case fatality rates for major bleeding were 1.6% and 10.4% in DOACs and in LMWH groups, respectively.^[25]^ Despite the favorable findings regarding treatment with DOACs in CAT, the clinical management of these patients is seriously challenging for several reasons. Firstly, cancer patients experience higher rates of both VTE recurrence and bleeding complications during anticoagulant therapy compared to non cancer patients.^[26]^ Secondly, cancer patients face various comorbidities such as renal impairment and thrombocytopenia. In addition, drug-drug interactions may arise due to the concomitant administration of anticancer and supportive therapy drugs. Although LMWH are still the standard of treatment for CAT, self-injections are burdensome for the patients and not cost-effective for society, particularly in active cancer given that it is expected long-term (>6 months) and can even require indefinite treatment until the cancer is no longer active.^[27]^ In this scenario, DOACs appear as a valid option. Beyond their proven efficacy and safety profile and the advantage of predictable effects, their ease of administration and fixed doses with no need for laboratory monitoring ensure adequate adherence to treatment.

The patient in this case had a primitive extratesticular seminoma. This is a very rare entity, accounting for about 3% of germ cell tumors. It occurs almost exclusively in males and the age of presentation is generally 20–35 years.^[28]^ More than one third of all malignant germ cell tumors are pure seminoma, one of the most chemo- and radiosensitive tumors.^[29]^ When treated, the prognosis is good, with a mean 5-year survival of 90%.^[30]^ Extragonadal germ cell tumors may present often in the mediastinal area and in a minority of cases in the retroperitoneal space.^[31]^ CAT frequency is around 8.1–26% in testicular germ cell tumors treated with platinum-based drugs, predominantly associated with advanced stage.^[32]^

Despite all the aforementioned improvements in the treatment of CAT, a *“dark side of the moon”* still remains, such as special populations or conditions that request challenging management.^[33]^Indeed, ULDVT is a rare site of the disease and relative evidence is poor. It is also called “Paget-von Schroetter disease” after the names of the researchers who reported and described the first 3 clinical cases.^[34]^ ULDVT includes the radial, ulnar, brachial, axillary, subclavian, internal jugular or brachiocephalic veins, while a thrombus in the cephalic and basilica veins is regarded as upper extremity superficial vein thrombosis.^[35]^ ULDVT accounts for 4–10% of all cases of DVT.^[36]^ It includes nonCA-ULDVT and catheter-related upper limb deep vein thrombosis (CA-ULDVT). The former condition may be idiopathic or due to cancer, estrogen use or other risk factors, while the latter is related to the presence of a catheter, pacemaker or port-system.^[37]^ ULDVT is subcategorized into primary and secondary according to etiology. Primary ULDVT represents one-third of all ULDVTs and includes idiopathic ULDVT, effort-related thrombosis and thrombosis related to the thoracic outlet syndrome. It is a rare condition (about 3/100,000/year) with a median age of 30 years. Secondary ULDVT is related to a triggering factor, such as indwelling lines (prevalence: 10–93%) or cancer (prevalence: 22–64%) with a mean age of presentation of 60 years. More than 40% of patients with ULDVT have concomitant cancer.^[38]^ ULDVT is an increasingly important condition, due to the growing rate of malignancy and also indwelling catheter use. Bleker et al analyzed 102 patients with ULDVT (41% had cancer), showing that 18% of the cancer population had recurrent VTE vs 7.5% in non cancer patients (adjusted hazard ratio AHR 2.2, 95%CI 0.6–8.2). The survival rate was 50% in cancer patients with ULDVT vs 60% in those without (AHR 0.8, 95%CI 0.4–1.4). Therefore, cancer patients presented a significant risk of recurrent DVT.^[39]^ ULDVT should not be undervalued; indeed, according to Di Nisio et al, after a first episode of ULDVT, patients may develop recurrent DVT in 3% up to 10% of cases and cancer patients showed a risk 2 to 3-fold higher than non cancer patients. In addition, the overall mortality rate was 24 to 35% after 1 year and the anticoagulant-related bleeding averaged 3.1 to 6.7%. As a result, due to the lack of RCTs on the treatment of ULDVT, current guidelines advocate recommendations mainly extrapolated from studies on the management of lower limb deep vein thrombosis (LLDVT), due to its higher incidence, and from studies on usual DVT sites.^[40]^ PE, the most serious of all VTE events, arises from LLDVT in 90% of patients as established in autopsy studies. Most ULDVT (87%) are catheter-related. IJDVT is the most common site of ULDVT, although noncatheter related forms are not well studied and the incidence of PE is not fully known.^[41]^ Notably, IJDVT usually follows intravenous drug abuse, prolonged central venous catheterization, invasive head and neck infections or head and neck trauma.^[42]^ Complications of ULDVT are mortality (10–50%, driven mainly by malignancy), post thrombotic syndrome and DVT recurrence. When we diagnose a ULDVT in a patient without an indwelling catheter, we should always rule out occult malignancy.^[43]^ According to the RIETE Registry, a prospective, multicenter, multinational registry, patients with nonCA-ULDVT (1100 patients, 29% had cancer)are less likely to present at baseline with PE than those with LEDVT (9.8% vs 25%, respectively). However, DVT recurrence during anticoagulant therapy was similar and 32% of patients with ULDVT died due to PE recurrences. Therefore, ULDVT should not be underestimated, since they are sometimes asymptomatic and patients should not be treated differently to those with LLDVT.^[44]^ A RIETE analysis documented that cancer and age were risk factors for bleeding and cancer was also a risk factor for DVT recurrence. Of note, only cancer was significantly associated with VTE recurrence, while other parameters such as immobility, recent surgery, thrombophilia, DVT history, effort thrombosis and hormonal therapy had no impact on the efficacy outcome.^[45]^ Nevertheless, the definition of CA-ULDVT was very heterogeneous. Therefore, decision-making on ULDVT treatment should be nuanced for each clinical subcategory as*“one size does not fit all”.*^[46]^ Indeed, in a prospective multicenter French cohort study (ONCOCIP) Decousus et al followed 3032 cancer patients with an implanted port for 12 months. VTE risk factors seemed to be different in patients with and without an indwelling catheter. Catheter-related DVT were mainly driven by mechanical causes such as the use of the cephalic vein for port implantation, whereas DVT unrelated to catheters were more likely a consequence of medical risk factors such as previous DVT events.^[47]^

In the clinical case we present, full dose enoxaparin was ineffective for the management of severe nonCA-ULDVT in a young male undergoing BEP chemotherapy. Mass effect, vascular compression from the tumor and, above all, the use of platinum compounds were all concomitant risk factors contributing to VTE persistence. This prothrombotic condition was responsible for the development of an extensive nonCA-ULDVT resistant to LMWH, which was resolved with the administration of edoxaban, a DOAC. The peculiarity of this case is the successful treatment with a DOAC in a patient with a rare form of cancer and a huge DVT in an unusual site. Data on the use of DOACs in this subpopulation of CAT are very scarce (anecdotal evidence, analysis of registries, small single-center studies), mainly conducted in non cancer patients and mostly evaluating rivaroxaban and not edoxaban, as in this case. In addition, these observational studies included heterogeneous populations in terms of thrombotic risk profile (different proportion of patients with cancer or indwelling lines), anticoagulant treatment (e.g., parenteral vs oral anticoagulant, different treatment durations) and outcome assessment. Indeed, DOACs have never been tested in patients with cancer and DVT in unusual sites, despite real-world emerging data showing their increasing use in active cancer patients. Indeed, in the GARFIELD-VTE Registry, 22.8% of patients with cancer received DOACs.^[48]^ In particular, the HOKUSAI-VTE CANCER trial, the only trial that examined the use of edoxaban compared to dalteparin in patients with CAT, included only patients with LLDVT or PE.^[20]^ The ADAM-VTE trial was the only head-to-head study to randomize also patients with ULDVT (15.3% of the included patients) to either apixaban or dalteparin.^[22]^ Nevetherless, although findings from direct comparison are missing for ULDVT, LMWH and DOACs are preferable to Vitamin K antagonists for the treatment of ULDVT.^[49]^ Vedovati et al found that DOACs are a feasible, effective and safe treatment option for patients with ULDVT. Of note, they reported in 188 patients (30% had cancer) that patients with cancer had higher rates of both VTE recurrence and bleeding compared to non cancer patients.^[50]^ To corroborate these results, a systematic review and meta-analysis conducted on a total of 1473 patients (56.1% had cancer) analyzed the efficacy and safety of anticoagulant treatment in ULDVT. The risk of DVT recurrence in patients with ULDVT during anticoagulant treatment was acceptably low, but the risk of bleeding was not negligible. These findings were consistent across the different subgroups of patients and the different anticoagulant agents.^[51]^ In this case report, we detected ULDVT by US, including compression ultrasound (CUS), color-Doppler and combined techniques, which is the imaging modality test of choice. Indeed, its sensitivity and specificity ranges from 81–97% and 93–96%, respectively.^[52]^ The negative predictive value of color-Doppler ± CUS is very high, reaching > 95% in most studies.^[53]^ To date, US imaging is the initial test of choice for the diagnosis of suspected ULDVT. Indeed, it offers several advantages over contrast venography, magnetic resonance imaging and computed tomography: good sensitivity and specificity, low risk due to the absence of radiation or contrast exposure, low cost and high availability.^[54]^

## 4. Conclusions

CAT is a common and dreaded complication that has a substantial impact on the quality of life and care in patients with cancer. Notable concerns refer to delays or discontinuation of anticancer drugs, leading to reduced effectiveness and oncological benefits. The strategy to govern proper anticoagulant treatment is challenging as prompt action is required. DOACs and LMWH are the mainstay of treatment to be continued while cancer is still at play. However, sometimes LMWH fail to solve huge VTE events, due to the underlying severe hypercoagulable state. DOACs such as edoxaban have proven effective and safe, as well as easy to use, even in cases with extended and marked DVT events; they are safe and not inferior to heparin in efficacy. In our opinion, DOACs play a key role in promoting a faster regression of thrombosis in active cancer compared to LMWH. Notably, ULDVT represents a considerable and more frequent burden and a serious entity not to be dismissed in terms of mortality and morbidity. However, it has never been systematically evaluated for anticoagulant treatment in cancer patients; its early recognition and treatment is of utmost importance to prevent complications. In addition, ad hoc trials properly designed in patients with nonCA-ULDVT and cancer are warranted, since published data are limited on this rare site of CAT which deserves dedicated investigations.

## Acknowledgments

We are grateful to Dr Angelo Virgilio Paradiso and Caroline Oakley for their helpful comments and careful reading of this manuscript.

## Author contributions

AMF, TL conceptualized the case report and wrote the original draft of the manuscript; AP, FG, and VL supervised the case report; GL and SO reviewed and edited the manuscript. All authors contributed to the article and read and approved the final manuscript.
